# Gut microbiota depletion from early adolescence alters anxiety and depression-related behaviours in male mice with Alzheimer-like disease

**DOI:** 10.1038/s41598-021-02231-0

**Published:** 2021-11-25

**Authors:** Belal Mosaferi, Yahya Jand, Ali-Akbar Salari

**Affiliations:** 1 Department of Basic Sciences, School of Nursing and Midwifery, Maragheh University of Medical Sciences, Maragheh, Iran; 2 Department of Pharmacology, School of Medicine, Tehran University of Medical Sciences, Tehran, Iran; 3 Salari Institute of Cognitive and Behavioral Disorders (SICBD), P.O. Box 31396-45999, Karaj, Alborz Iran

**Keywords:** Anxiety, Depression, Stress and resilience

## Abstract

The gut-microbiota–brain axis plays an important role in stress-related disorders, and dysfunction of this complex bidirectional system is associated with Alzheimer’s disease. This study aimed to assess the idea that whether gut microbiota depletion from early adolescence can alter anxiety- and depression-related behaviours in adult mice with or without Alzheimer-like disease. Male C57BL/6 mice were treated with an antibiotic cocktail from weaning to adulthood. Adult mice received an intracerebroventricular injection of amyloid-beta (Aβ)1–42, and were subjected to anxiety and depression tests. We measured, brain malondialdehyde and glutathione following anxiety tests, and assessed brain oxytocin and the hypothalamic–pituitary–adrenal (HPA) axis function by measuring adrenocorticotrophic hormone (ACTH) and corticosterone following depression tests. Healthy antibiotic-treated mice displayed significant decreases in anxiety-like behaviours, whereas they did not show any alterations in depression-like behaviours and HPA axis function. Antibiotic treatment from early adolescence prevented the development of anxiety- and depression-related behaviours, oxidative stress and HPA axis dysregulation in Alzheimer-induced mice. Antibiotic treatment increased oxytocin in the brain of healthy but not Alzheimer-induced mice. Taken together, these findings suggest that gut microbiota depletion following antibiotic treatment from early adolescence might profoundly affect anxiety- and depression-related behaviours, and HPA axis function in adult mice with Alzheimer-like disease.

## Introduction

In the past two decades, clinical and experimental studies have demonstrated that gut microbiota plays a crucial role in brain function and behaviour^1^. There are bidirectional links and communication systems between the gut microbiota and the central nervous system, referred to as the “gut microbiota–brain axis”^2^. This bidirectional communication occurs through multiple pathways involving endocrine, immune and neural systems^3^. It is now well documented that gut microbiota is associated with the pathogenesis of several neurodegenerative and neuropsychiatric disorders such as Alzheimer's disease (AD), anxiety and major depression^4–6^. To perform research on brain disorders, preclinical studies mainly rely on animal models^7^. In this regard, the majority of gut microbiota studies use germ-free and antibiotic animal models. The antibiotic treatment model has received considerable attention due to its efficacy in depleting the gut microbiota for a long-time period at different developmental stages^5,8^. This animal model can be more controlled than the germ-free mice which have underdeveloped immune systems, different metabolic activities, and more permeable blood–brain barrier (BBB)^9–11^. Based on this evidence, the germ-free findings have to be interpreted with caution as the findings might not be transferable to humans. Gut microbiota depletion induced by antibiotic treatment from early adolescence (a critical developmental window of vulnerability to neuropsychiatric disorders) was shown to be associated with increased anxiety- and depression-related behaviours later in life^5,8^.

Alzheimer's disease is one of the most complex neurodegenerative disorders affecting more than 45 million people worldwide^6^. The most important pathological hallmark of AD is amyloid-beta (Aβ) peptide deposition in brain tissue^12^. In recent years, there has been an intensified effort to understand the complexity of the relationship between gut microbiota and AD using germ-free and antibiotic models^13–15^. Alterations in the diversity and composition of gut microbial ecosystems are associated with anxiety-related disorders and major depression. Substantial research has demonstrated that rodents treated with antibiotics or bred under germ-free conditions display significant changes in anxiety- and depression-related behaviours^1,5,8,16–21^. Furthermore, clinical studies suggest considerable comorbidity between AD and anxiety or depression^22,23^. These findings are broadly supported by the animal studies linking brain Aβ administration to anxiety- and depression-like behaviours^24–26^ Although there is a high prevalence of anxiety and depression in AD patients, no previous study has investigated the effects of gut microbiota depletion from early adolescence on anxiety- and depression-related symptoms in adult mice with Alzheimer-like disease.

Understanding the neurobiological mechanisms underpinning brain and behavioural abnormalities induced by gut microbiota manipulation is now a key research priority. Previous studies have shown a relationship between gut microbiota and oxidative stress^27,28^. There is homeostasis between oxidant and antioxidant markers in the body under normal physiological conditions^29^. Given that oxidative stress is involved in both AD and anxiety disorder^30,31^, and that psychological distress and physical stress can reduce antioxidant factors and increase oxidative stress in the brain^30^; it seems possible that oxidative stress might be a player in the regulation of anxiety-like behaviour following gut microbiota depletion in AD-induced mice. Furthermore, studies over the past 10 years have provided important information about the effects of intestinal microbiota on the hypothalamic–pituitary–adrenal (HPA) axis^32^, and the oxytocinergic system^8,33,34^. The link between the HPA axis and depression has been well documented in both human and animal studies^25,35,36^. Oxytocin is a peptide hormone with a key role in social attraction, affiliative behaviour and bonding, which can play a key role in the development of depression^37,38^. This neuropeptide also interacts with the HPA axis system to regulate depression-related behaviours^25,36,38–42^. Accordingly, the relationship between antibiotic-induced gut microbiota depletion and these hormonal systems might be involved in the pathogenies of depression-related behaviours in AD-induced mice.

Therefore, this study was undertaken to investigate whether antibiotic-induced gut microbiota depletion from early adolescence can affect anxiety- and depression-related behaviours in adult male C57BL/6 mice with or without Alzheimer-like disease. The relationship between anxiety-like behaviour and oxidative stress markers (malondialdehyde and glutathione) in the brain, and between depression-like behaviour and brain oxytocin or HPA axis were evaluated. Adrenocorticotrophic hormone (ACTH) and corticosterone were also measured as indexes of HPA axis function.

## Results

### Anxiety tests

#### Open field

**Figure 1:**
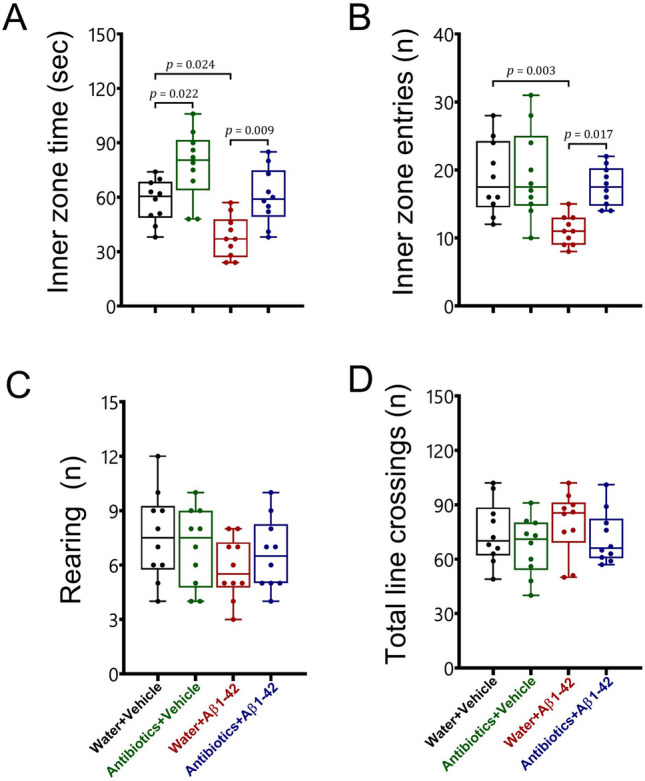
The effects of antibiotic treatment alone or in combination with Aβ1–42 treatment on anxiety-related behaviour in male C57BL6 mice. To assess anxiety-related behaviour (**A** inner zone time; **B** inner zone entries) and locomotor activity (**C** rearing; **D** total line crossings), animals were subjected to the open field test. Data are expressed as mean ± SEM (n = 10). Antibiotic treatment significantly increased the inner zone time [*p* = 0.022], but it did not affect the other parameters [*p* > 0.05] compared to the water + vehicle mice. On the other hand, Aβ 1–42 administration in water-treated mice significantly decreased the inner zone time [*p* = 0.024] and entries [*p* = 0.003] compared to the water + vehicle group. However, Aβ 1–42 injection could not affect the inner zone time and entries [*p* > 0.05] in antibiotic-treated mice compared to the water + vehicle or antibiotics + vehicle groups. Besides, significant differences in all behavioural parameters in the open field test were not observed between antibiotics + Aβ 1–42 mice and water + vehicle group [*p* > 0.05]. Moreover, there are significant differences in the inner zone time [*p* = 0.009] and entries [*p* = 0.017] between water + Aβ 1–42-treated mice and antibiotics + Aβ 1–42-treated animals. Furthermore, both antibiotic and Aβ 1–42 treatments did not change [*p* > 0.05] the rearing and the total line crossings in the open field test.

To measure anxiety-like behaviour and general locomotor activity, animals were exposed to the open field test (Fig. 1). Antibiotic-treated healthy mice displayed a significant reduction in anxiety-related behaviour in the open field test. Alzheimer induction also increased anxiety-related behaviour in mice. Interestingly, the antibiotic treatment prevented the development of anxiety-related behaviour in male C57BL/6 mice with Alzheimer-like disease. No significant changes were observed in the locomotor activity. A significant interaction was detected between antibiotic treatment and Aβ 1–42 administration for the inner zone entries [F_1,36_ = 4.35, *p* = 0.044], while there was no such interaction [F_1,36_ < 1.2, *p* > 0.05] for the inner zone time, rearing, and total line crossings. There were significant main effects of antibiotic treatment and Aβ 1–42 administration for the inner zone time [F_1,36_ = 20.75, *p* < 0.001; F_1,36_ = 15.97, *p* < 0.001, respectively] and entries [F_1,36_ = 5.57, *p* = 0.024; F_1,36_ = 10.57, *p* = 0.002, respectively], but not for rearing and total line crossings [F_1,36_ < 2.8, *p* > 0.05].

#### Light–dark box

**Figure 2:**
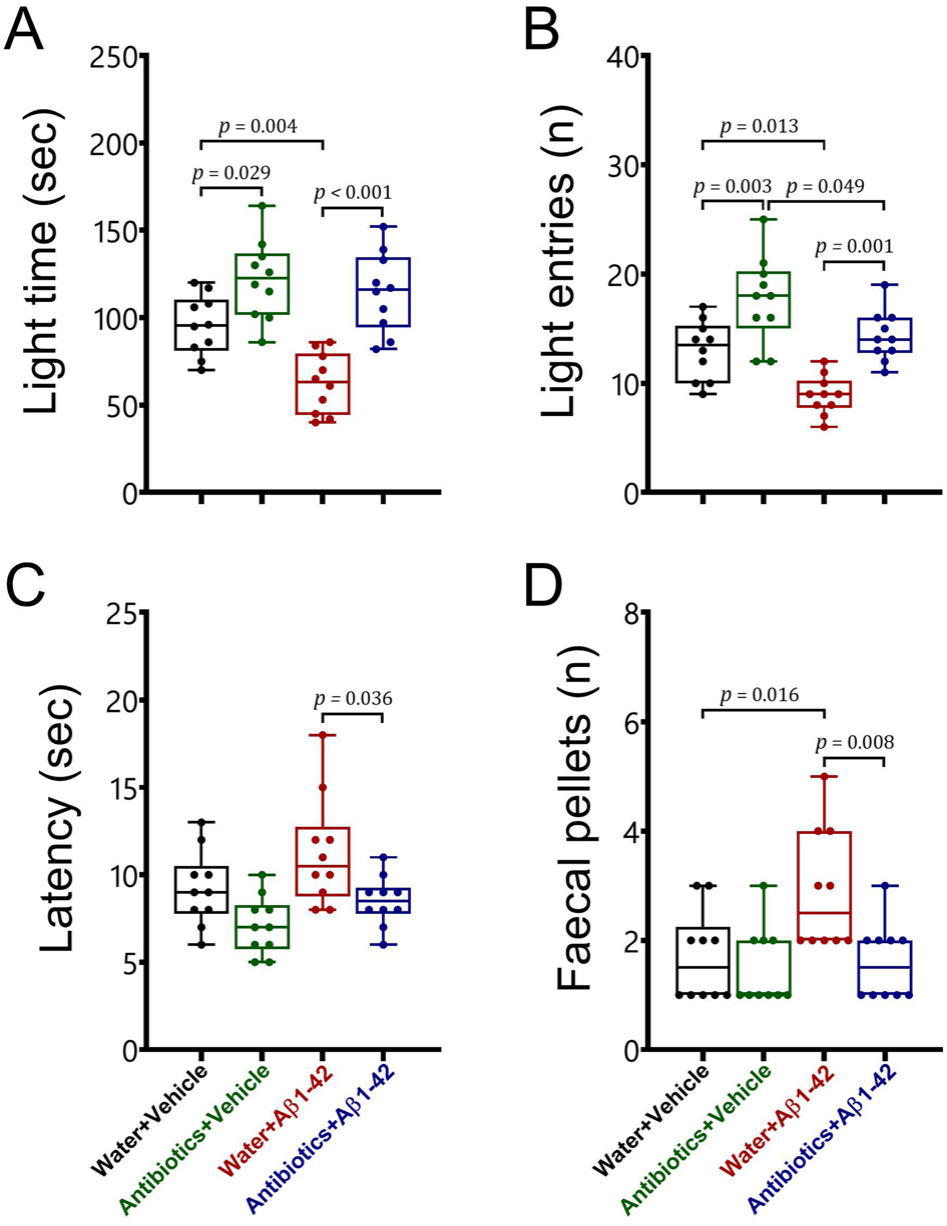
The effects of antibiotic treatment alone or in combination with Aβ1–42 treatment on anxiety-related behaviour in male C57BL6 mice. To examine anxiety-related behaviour, animals were subjected to the light–dark box (**A** light time; **B** light entries; **C** latency; **D** faecal pellets). Data are expressed as mean ± SEM (n = 10). Antibiotic treatment significantly increased the time spent [*p* = 0.029] and the number of entries [*p* = 0.003] in the light area, whereas it did not affect the latency and faecal pellets [*p* > 0.05] in the light–dark box, as compared to the water + vehicle group. On the other hand, Aβ 1–42 administration significantly reduced the light time [*p* = 0.004] and entries [*p* = 0.013], and increased fecal pellets [*p* = 0.016; **D**] in water-treated mice, as compared to the water + vehicle group. In addition, Aβ 1–42 treatment significantly decreased the light entries [*p* = 0.049], but not the other behavioral parameters [*p* > 0.05] in antibiotic-treated mice relative to the antibiotics + vehicle group. However, we did not detect any significant differences in all behavioural parameters in the light–dark task between antibiotics + Aβ 1–42 mice and water + vehicle group [*p* > 0.05]. On the other side, significant differences in the light time [*p* < 0.001], the light entries [*p* = 0.001], the latency [*p* = 0.036], and faecal pellets [*p* = 0.008] were found between water mice treated with Aβ 1–42 and antibiotics + Aβ 1–42-treated mice.

To assess anxiety-related behaviour, the mice were also subjected to the light dark box (Fig. 2). We found that antibiotic treatment prevented the development of anxiety-related behaviours in both healthy and Alzheimer-induced mice in the light–dark box. Significant interactions were observed between antibiotic treatment and Aβ1–42 administration for the light time [F_1,36_ = 4.13, *p* = 0.049] and fecal pellets [F_1,36_ = 4.2, *p* = 0.048]. However, no significant interactions [F_1,36_ < 0.25, *p* > 0.05] were detected for the light entries and the latency. We also found significant main effects of antibiotic treatment and Aβ 1–42 administration for the light time [F_1,36_ = 38.01, *p* < 0.001; F_1,36_ = 10.11, *p* = 0.003, respectively], light entries [F_1,36_ = 32.2, *p* < 0.001; F_1,36_ = 17.75, *p* < 0.001, respectively], the latency [F_1,36_ = 12.34, *p* = 0.001; F_1,36_ = 6.29, *p* = 0.017, respectively], and fecal pellets [F_1,36_ = 7.81, *p* = 0.008; F_1,36_ = 5.87, *p* = 0.021, respectively].

#### Zero maze

**Figure 3:**
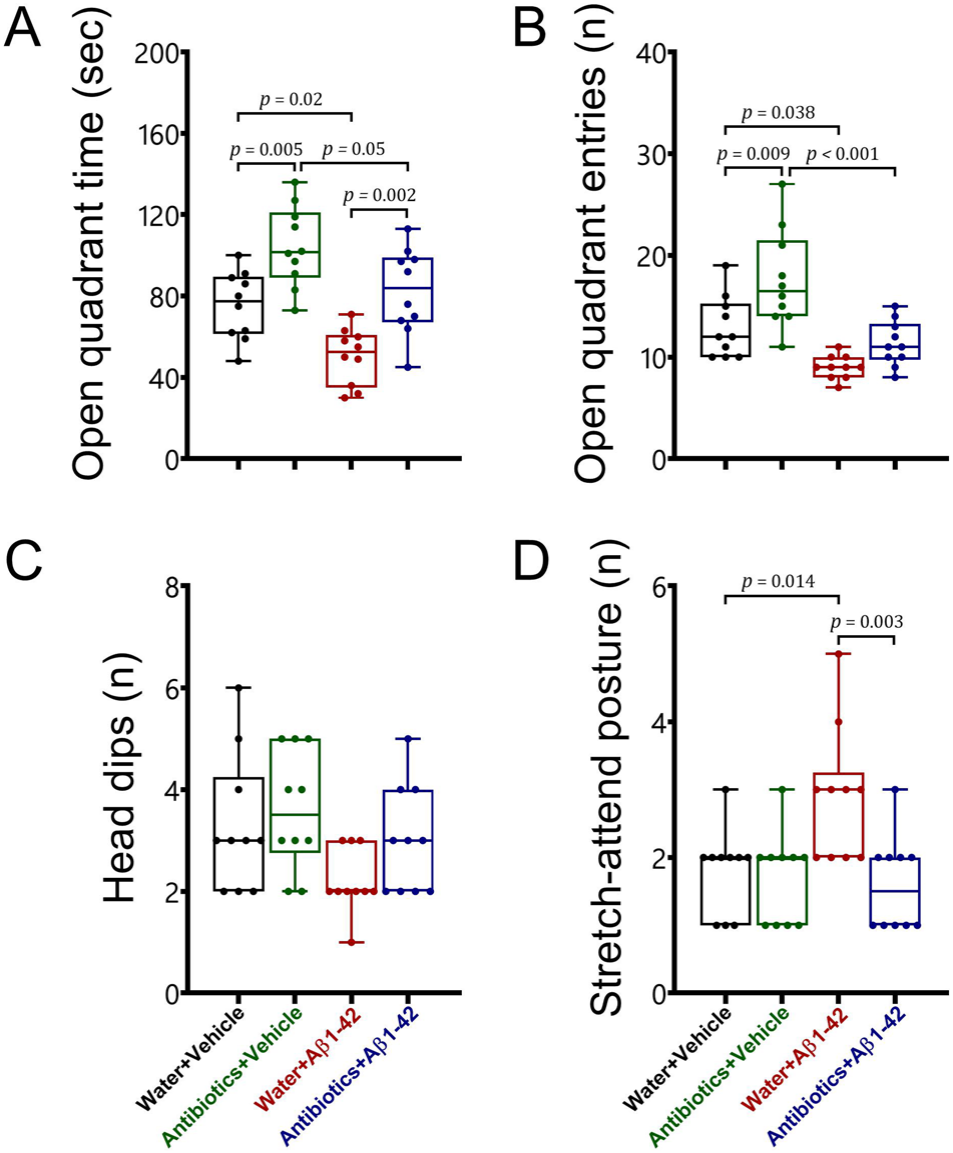
The effects of antibiotic treatment alone or in combination with Aβ1–42 treatment on anxiety-related behaviour in male C57BL6 mice. To evaluate anxiety-related behaviour, animals were subjected to the zero maze (**A** open quadrant time; **B** open quadrant entries; **C** head dips; **D** stretch-attend posture). Data are expressed as mean ± SEM (n = 10). Antibiotic-treated mice exhibited a significant increase in the open quadrant time [*p* = 0.005] and entries [*p* = 0.009], while no alterations [*p* > 0.05] were found in the head dips and stretch-attend postures following antibiotic treatment, as compared to the water + vehicle group. On the other side, Aβ 1–42 injection significantly decreased the time spent [*p* = 0.02] and the number of entries [*p* = 0.038] in the open quadrants and increased the stretch-attend postures [*p* = 0.014] in water-treated mice in comparison with the water + vehicle group. In addition, antibiotics mice treated with Aβ 1–42 displayed a decrease in the open quadrant time [*p* = 0.05] and entries [*p* < 0.001] as compared to the antibiotics + vehicle group. However, there was no significant difference in all parameters in the zero maze between antibiotics + Aβ 1–42 mice and water + vehicle group [*p* > 0.05]. Furthermore, we found significant differences in the open quadrant time [*p* = 0.002] and the stretch-attend postures [*p* = 0.003] between water + Aβ 1–42 group and antibiotics + Aβ 1–42-treated mice.

To evaluate anxiety-related behaviour in animals, the zero maze was also used (Fig. 3). Statistical analysis revealed that sustained antibiotic treatment prevented the development of anxiety-related behaviour in both healthy and Alzheimer-induced mice in the zero maze. We found a significant interaction between antibiotic treatment and Aβ 1–42 administration for the stretch-attend postures [F_1,36_ = 6.17, *p* = 0.018]. However, there were no significant interactions [F_1,36_ < 1.5, *p* > 0.05] for the open quadrant time, entries, and head dips. There were also main effects of antibiotic treatment or Aβ 1–42 administration for the open quadrant time [F_1,36_ = 28.57, *p* < 0.001; F_1,36_ = 16.69, *p* < 0.001, respectively], the open quadrant entries [F_1,36_ = 12.69, *p* = 0.001; F_1,36_ = 26.95, *p* < 0.001, respectively], head dips [F_1,36_ = 2.58, *p* > 0.05; F_1,36_ = 6.17, *p* = 0.018, respectively], and stretch-attend postures [F_1,36_ = 8.4, *p* = 0.006; F_1,36_ = 4.28, *p* = 0.046, respectively].

### Oxidative stress and antioxidant markers

**Figure 4:**
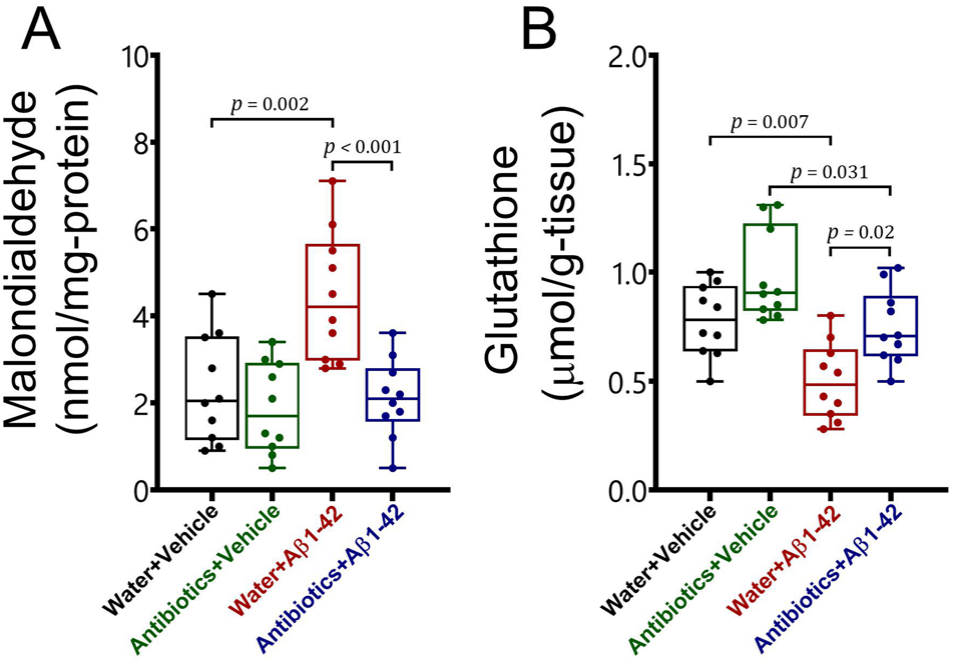
The effects of antibiotic treatment alone or in combination with Aβ1–42 treatment on oxidative stress and antioxidant markers (**A** malondialdehyde; **B** glutathione) in the brain of male C57BL6 mice. Data are expressed as mean ± SEM (n = 10). Antibiotic treatment alone did not affect malondialdehyde and glutathione levels in the brain of mice [*p* > 0.05] as compared to the water + vehicle group. On the other hand, Aβ 1–42 treatment significantly increased malondialdehyde [*p* = 0.002] and decreased glutathione [*p* = 0.007] in water-treated mice as compared to the water + vehicle group. In addition, Aβ 1–42 administration significantly reduced glutathione [*p* = 0.031], whereas did not affect malondialdehyde levels [*p* > 0.05] in the brain of antibiotic-treated mice as compared to the antibiotics + vehicle group. However, no significant differences in these markers existed between antibiotics + Aβ 1–42 and water + vehicle groups [*p* > 0.05]. Furthermore, there were significant differences in malondialdehyde [*p* < 0.001] and glutathione [*p* = 0.02] levels in the brain between water + Aβ 1–42-treated animals and antibiotics + Aβ 1–42 mice.

We measured malondialdehyde and glutathione levels in the whole brain to evaluate a possible association between anxiety-like behaviours and oxidative stress or antioxidant system (Fig. 4). The findings showed that antibiotic treatment from early adolescence significantly prevented the development of oxidative stress in the brain of Alzheimer-induced mice. We did not find any alterations in oxidative and antioxidant markers following antibiotic treatment in the brain of healthy mice. The analysis revealed a significant interaction between antibiotic treatment and Aβ 1–42 administration for malondialdehyde [F_1,36_ = 6.45, *p* = 0.016] but not for glutathione [F_1,36_ = 0.16, *p* > 0.05] levels in the brain. Besides, we detected main effects of antibiotic treatment and Aβ 1–42 treatment for both malondialdehyde [F_1,36_ = 13.82, *p* = 0.001; F_1,36_ = 9.96, *p* = 0.003, respectively], and glutathione [F_1,36_ = 15.66, *p* < 0.001; F_1,36_ = 20.27, *p* < 0.001, respectively].

### Correlations between anxiety tests and brain measurements

**Figure 5:**
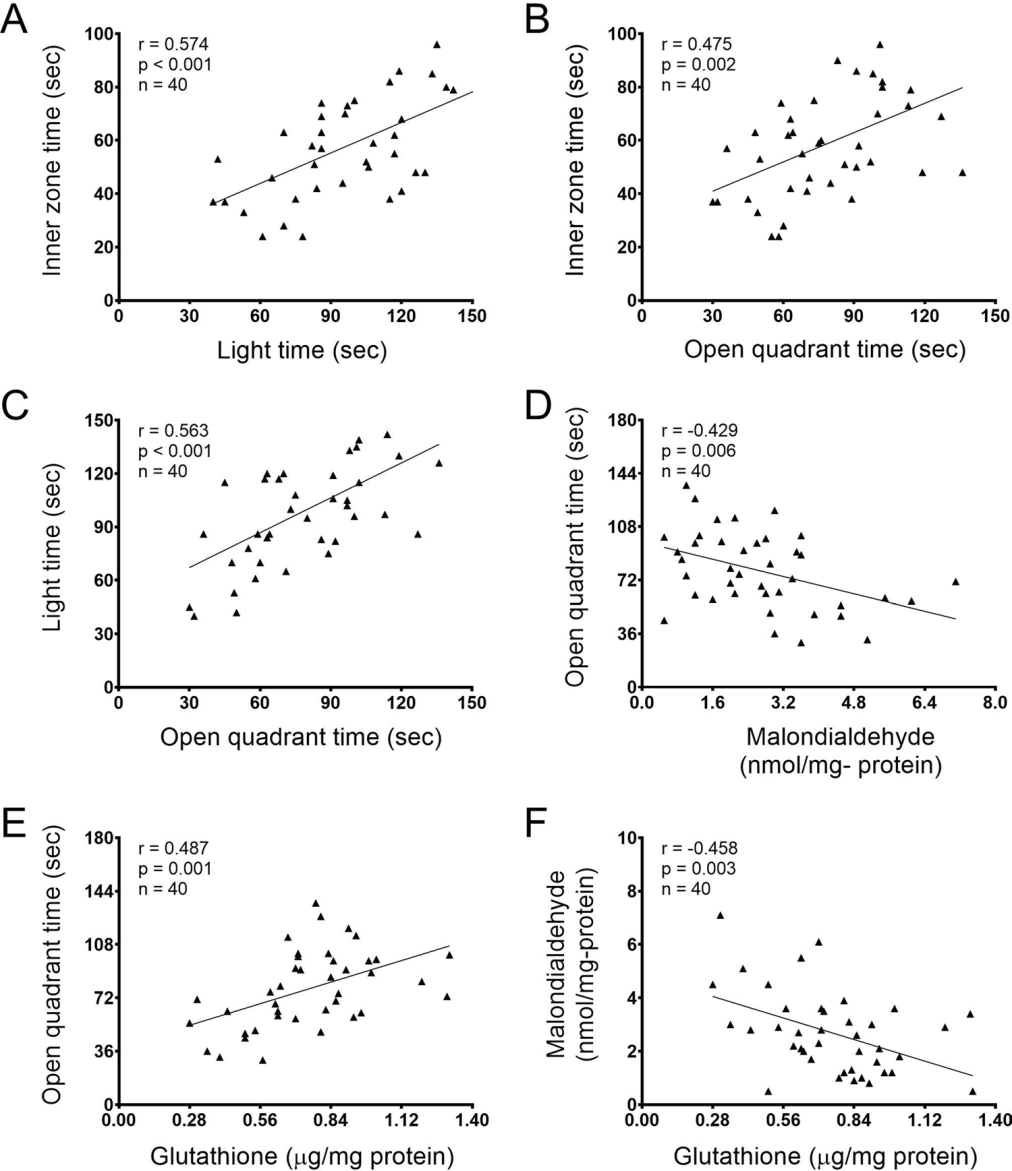
Pearson correlations between anxiety tests and/or oxidative stress/antioxidant markers. Correlation coefficient: r; the number of animals: n; p-value: p.

We found positive correlations between the inner zone time (open field) and the light time (light–dark box) [*r* = 0.574, *p* < 0.001; Fig. 5A], between the inner zone time (open field) and the open quadrant time (zero maze) [*r* = 0.475, *p* = 0.002; Fig. 5B], between the light time (light–dark box) and the open quadrant time (zero maze) [*r* = 0.563, *p* < 0.001; Fig. 5C], and between the open quadrant time (zero maze) and glutathione in the brain [*r* = 0.487, *p* = 0.001; Fig. 5E]. There were also negative correlations between the open quadrant time (zero maze) and malondialdehyde in the brain [*r* = − 0.429, *p* = 0.006; Fig. 5D], and between malondialdehyde and glutathione [*r* = − 0.458, *p* = 0.003; Fig. 5F] in the brain.

### Depression tests, HPA axis and oxytocin

#### Sucrose preference test

**Figure 6:**
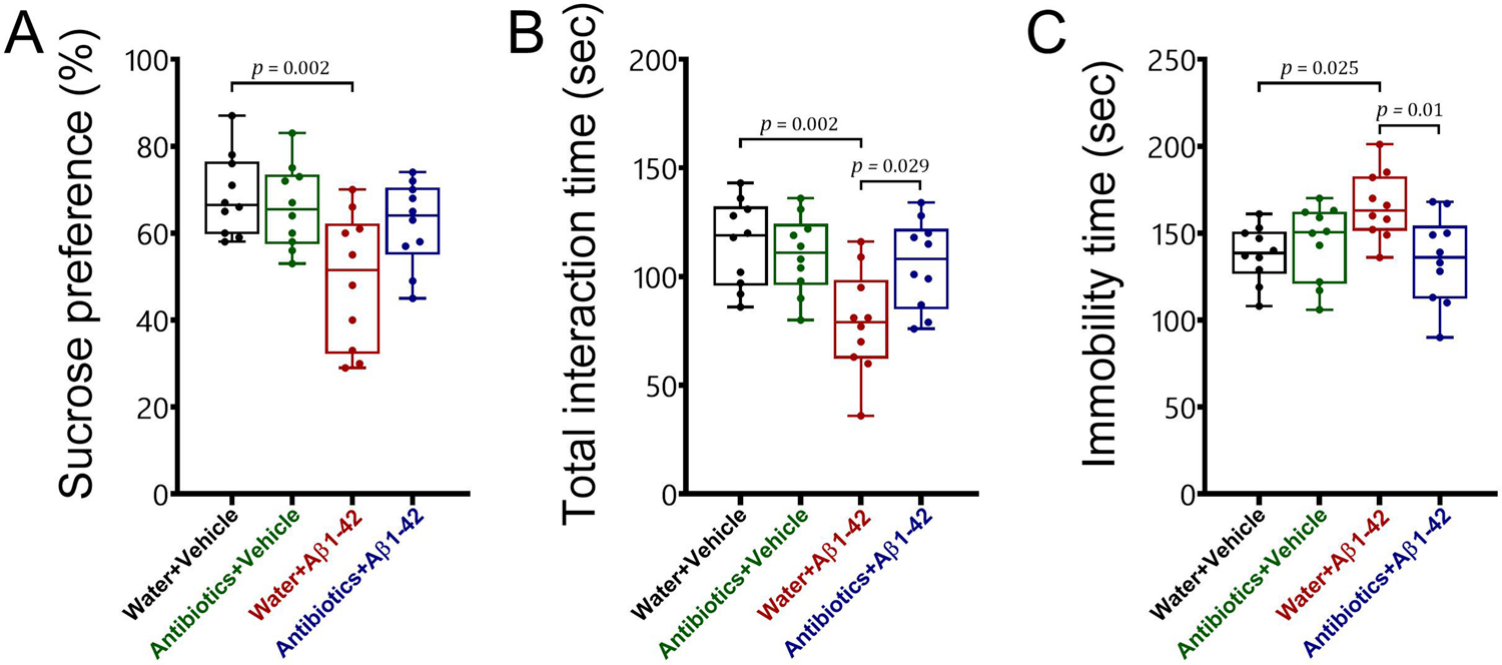
The effects of antibiotic treatment alone or in combination with Aβ1–42 treatment on depression-related behaviours in male C57BL6 mice. To assess depression-related behaviour, animals were subjected to sucrose preference (**A**), social interaction (**B**; total interaction time) and forced swim (**C**; immobility time) tests. Data are expressed as mean ± SEM (n = 10). Antibiotic treatment did not change [*p* > 0.05] the percentage of sucrose preference, total interaction time, and immobility time in mice relative to the water + vehicle group. We also found that Aβ 1–42 administration significantly decreased the percentage of sucrose preference [*p* = 0.002] and the total interaction time [*p* = 0.002] but increased the immobility time [*p* = 0.025] in water-treated mice as compared to the water + vehicle animals. In addition, there were significant differences in the total interaction time [*p* = 0.029] and the immobility time [*p* = 0.01] between water + Aβ 1–42 and antibiotics + Aβ 1–42 groups. However, antibiotic treatment could not affect the sucrose preference [*p* > 0.05] in antibiotic-treated mice as compared to both the antibiotics + vehicle and water + vehicle groups. We also did not find any significant differences [*p* > 0.05] in sucrose preference, the total interaction time and the immobility time between antibiotics mice treated with Aβ 1–42 and the antibiotics + vehicle- or water + vehicle-treated animals.

To assess anhedonia-like symptoms in mice, the sucrose preference test was used (Fig. 6A). Antibiotic-treated mice exhibited significant resistance to Alzheimer-induced anhedonia-like behaviour in the sucrose preference test. No significant change in anhedonia-like behaviour following antibiotic treatment was observed in healthy mice. The statistical analysis revealed a significant interaction between antibiotic treatment and Aβ 1–42 administration for the percentage of sucrose preference [F_1,36_ = 4.7, *p* = 0.037]. We also found a main effect for Aβ 1–42 administration [F_1,36_ = 10.8, *p* = 0.002] but not for antibiotic treatment [F_1,36_ = 2.07, *p* > 0.05].

#### Social interaction test

To evaluate social behaviour, the mice were subjected to the social interaction test (Fig. 6B). Our findings indicate that sustained antibiotic treatment prevented the impairment of social interaction in Alzheimer-induced mice. However, it did not affect social behaviour in healthy mice. A statistically significant interaction was identified between antibiotic treatment and Aβ 1–42 administration for the total interaction time [F_1,36_ = 6.05, *p* = 0.019]. There was also a main effect for Aβ 1–42 injection [F_1,36_ = 9.93, *p* = 0.003], while no significant main effect was observed for antibiotic treatment [F_1,36_ = 2.8, *p* > 0.05].

#### Forced swim test

To assess behavioural despair, the animals were subjected to the forced swim test (Fig. 6C). Statistical analysis revealed that sustained antibiotic treatment prevented the development of behavioural despair in Alzheimer-induced mice, whereas antibiotic treatment did not alter behavioural despair in healthy mice. A significant interaction was found between antibiotic treatment and Aβ 1–42 administration for the immobility time [F_1,36_ = 8.04, *p* = 0.007]. However, there were no main effects for both antibiotic [F_1,36_ = 3.54, *p* > 0.05] and Aβ 1–42 treatments [F_1,36_ = 1.91, *p* > 0.05].

#### HPA axis function

**Figure 7:**
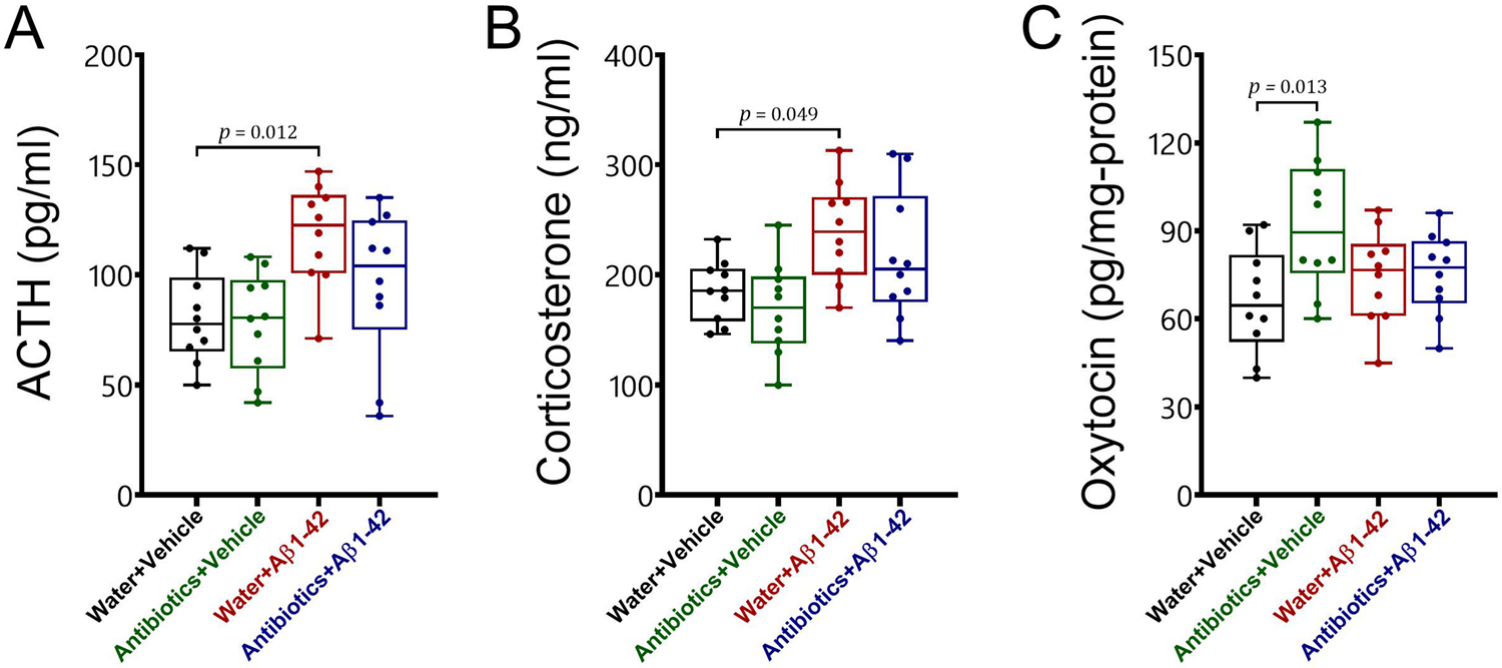
The effects of antibiotic treatment alone or in combination with Aβ1–42 treatment on HPA axis function (**A** ACTH; **B** corticosterone) and brain oxytocin (**C**) in male C57BL6 mice. Data are expressed as mean ± SEM (n = 10). Antibiotic treatment significantly increased brain oxytocin [*p* = 0.013] but did not alter [*p* > 0.05] ACTH and corticosterone levels in the serum of mice as compared to the water + vehicle group. In addition, Aβ 1–42 administration significantly increased ACTH [*p* = 0.012] and corticosterone levels [*p* = 0.049] but not brain oxytocin [*p* > 0.05] in water-treated mice as compared to the water + vehicle group. Furthermore, no significant differences [*p* > 0.05] in ACTH, corticosterone, and oxytocin levels were observed between antibiotics + Aβ 1–42 mice and antibiotics + vehicle or water + vehicle groups.

To evaluate HPA axis activity, we measured ACTH and corticosterone levels in serum (Fig. 7A,B). The results revealed that antibiotic treatment prevented the development of HPA axis hyperactivity in Alzheimer-induced mice. No significant alteration was observed in HPA axis function following antibiotic treatment in healthy mice. There was no significant interaction between antibiotic treatment and Aβ 1–42 administration for both ACTH [F_1,36_ = 1.5, *p* > 0.05] and corticosterone [F_1,36_ = 0.05, *p* > 0.05]. A significant main effect was found for Aβ 1–42 administration [ACTH: F_1,36_ = 11.48, *p* = 0.002; corticosterone: F_1,36_ = 12.9, *p* = 0.001] but not for antibiotic treatment [ACTH: F_1,36_ = 2.15, *p* > 0.05; corticosterone: F_1,36_ = 1.87, *p* > 0.05].

#### Oxytocin

We found that sustained antibiotic treatment resulted in a significant increase in oxytocin levels in the brain of healthy but not Alzheimer-induced mice (Fig. 7C). A significant interaction was identified between antibiotic treatment and Aβ 1–42 administration for oxytocin levels in the brain [F_1,36_ = 4.83, *p* = 0.034]. There was also a main effect for antibiotic treatment [F_1,36_ = 5.65, *p* = 0.023] but not for Aβ 1–42 administration [F_1,36_ = 0.53, *p* > 0.05].

### Correlations between depression tests and HPA axis or oxytocin

**Figure 8:**
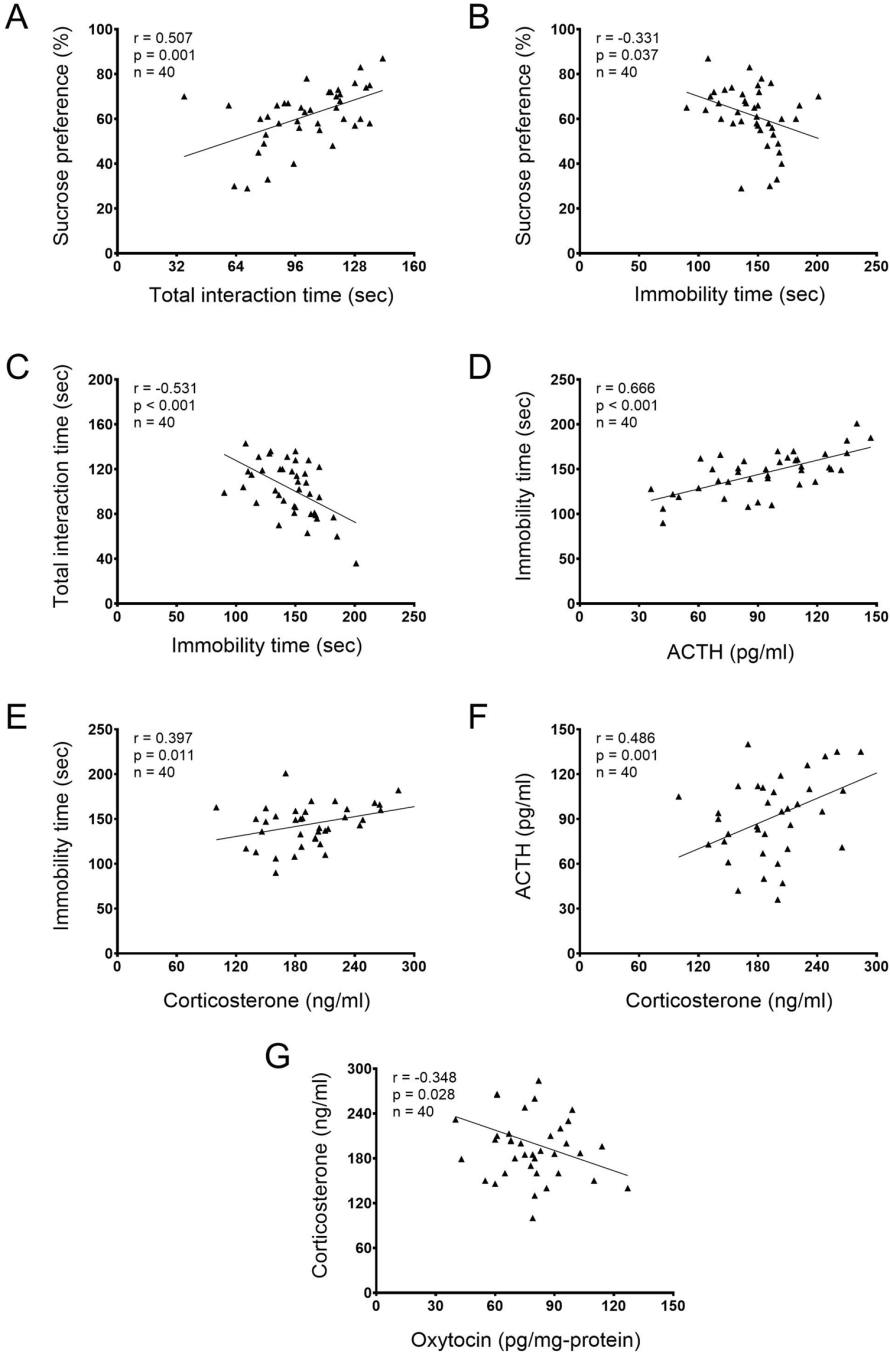
Pearson correlations between depression tests and HPA axis function or oxytocin. Correlation coefficient: r; the number of animals: n; p-value: p.

There were significant positive correlations between sucrose preference and the total interaction time (social behavior) [*r* = 0.507, *p* = 0.001; Fig. 8A], between the immobility time (forced swim test) and ACTH [*r* = 0.666, *p* < 0.001; Fig. 8D], between the immobility time (forced swim test) and corticosterone [*r* = 0.397, *p* = 0.011; Fig. 8E], and between ACTH and corticosterone [*r* = 0.486, *p* = 0.001; Fig. 8F].

We also found significant negative correlations between sucrose preference and the immobility time (forced swim test) [*r* = − 0.331, *p* = 0.037; Fig. 8B], between the total interaction time (social behavior) and the immobility time (forced swim test) [*r* = − 0.531, *p* < 0.001; Fig. 8C], and between serum corticosterone and oxytocin level in the brain [*r* = − 0.348, *p* = 0.028; Fig. 8G].

## Discussion

Antibiotic-treated male C57BL/6 mice exhibited significant decreases in anxiety-related symptoms in all behavioural tests including open field, light–dark box, and zero maze. Although Aβ administration increased anxiety-like behaviours in water-treated mice, it did not induce these behavioural abnormalities in antibiotic-treated mice. Previous studies have reported that Aβ administration increases anxiety-related behaviours in rodents^25,26,43–47^. However, to date, there are no available reports on the gut microbiota depletion and resistant to Aβ-induced anxiety-like behaviour. In support of our findings, two studies reported that antibiotic treatment in male NIH mice during adolescence or adulthood significantly decreased anxiety-related symptoms in the light–dark box^8,48^. Consistent with these antibiotic models, male^1,49^ and female^18^ germ-free mice were shown to have less anxious-like behaviour in the elevated plus-maze, light–dark box and open field test. In contrast to this evidence, male F344 rats housed in germ-free conditions exhibited increased anxiety-like behaviour in the open field test^19^. Furthermore, adolescent antibiotic treatment was shown to considerably increase anxiety-like behaviour in adult male C57Bl/6OlaHsd mice, whereas no significant changes in adult anxiety-like behaviour were observed following adult antibiotic treatment in male C57Bl/6OlaHsd or BALB/c mice^20,21^. When we used this antibiotic treatment protocol in female C57BL/6 mice in another study, we found that gut microbiota depletion increased anxiety-like behaviour in mice with or without experimental autoimmune encephalomyelitis (EAE)^5^. Sex might play a role in the development of anxiety-like behaviour following antibiotic treatment later in life. What can be concluded from these findings is that different factors such as the microbiota deficient model, the type of antibiotics, treatment duration, species, strain and sex of rodents might differently affect anxiety-like behaviour in mice with or without Alzheimer-like disease. For instance, the blood–brain barrier is more permeable in germ-free mice^9^, and Cryan’s lab suggested that antibiotics treatment from early adolescence can represent a more amenable alternative for germ-free mice in the assessment of microbiota modulation of behaviour^8^.

Oxidative stress-related markers including malondialdehyde and glutathione were also measured in the brain of mice to investigate the possible mechanism of antibiotic treatment in anxiety-like behaviour^30,50,51^. Antibiotic treatment did not alter these factors in the brain of vehicle-treated mice, whereas Aβ administration significantly increased malondialdehyde and decreased glutathione in the brain of water-treated mice. Interestingly, there were significant differences in these factors between water and antibiotics mice treated with Aβ. In fact, no significant alterations in oxidant and antioxidant markers were observed following Aβ treatment in antibiotic-treated mice. This means antibiotic-treated mice were resistant to Aβ-induced alteration in brain oxidative stress. It has been well recognized that Aβ induces oxidative stress in the brain^52–54^. And, antibiotic treatment can effectively reduce brain oxidative stress in rodents^55,56^. Given that oxidative stress plays an important role in anxiety disorders and Alzheimer’s disease, and it could be influenced by gut microbiota manipulations^27,28,57,58^; therefore, it seems that gut microbiota depletion from early adolescence might prevent anxiety-like behaviour by reducing brain oxidative stress in Aβ-treated mice. Although there are correlations between anxiety-related parameters and oxidant/antioxidant markers in the brain, further studies are required to assess whether antioxidant drugs or probiotic products with antioxidant impacts can reduce anxiety-like behaviours in preclinical and clinical Alzheimer studies.

In the current study, we did not observe any alterations in depression-related symptoms in male antibiotic-treated mice in all behavioural tests. As expected, Aβ-treated mice displayed depressive-like behaviour by decreasing sucrose consumption and social behaviour and increasing behavioural despair^24,44,45,59,60^. By contrast, antibiotic treatment from early adolescence prevented the development of AD-induced depression-related behaviours in mice. However, we have previously reported that female C57BL/6 mice treated with antibiotics from early adolescence exhibited a more susceptibility to depression-like behaviour following EAE induction, no significant changes in depression-related symptoms were also found in antibiotic-treated mice without EAE^5^. Along with these findings, several studies have revealed that gut microbiota depletion in male antibiotics-treated Sprague Dawley rats and male germ-free mice result in increased depressive-like behaviour in the forced swim test^11,61,62^. It can thus be suggested that antibiotic treatment from early adolescence to adulthood may make male C57Bl/6 mice resistant to depression-like behaviours. These results provide further support for the idea that gut microbiota has a critical role in the regulation of depression-related disorders^63–67^. To develop a full picture of gut microbiota role during adolescence on brain and behaviour functions later in life, additional studies with more focus on a specific period are therefore recommended.

Interestingly, antibiotic treatment alone did not change ACTH and corticosterone levels in mice, but Aβ administration increased these hormones in water-treated mice. What makes this study more valuable is that we did not observe any notable alterations in both ACTH and corticosterone levels following Aβ administration in antibiotic-treated mice. The correlations between HPA axis-related hormones and depression-like behaviours demonstrate that the HPA system could play an important role in the development of depressive-like behaviour following gut microbiota manipulations^32,68,69^. For instance, the dysregulation of the HPA axis system in germ-free mice was confirmed by increasing plasma ACTH and corticosterone levels^70,71^. In accordance with this evidence, antibiotic treatment was shown to increase corticosterone levels in early adolescence and adulthood in mice^72^. A likely explanation for this difference between these studies and our results could be the oxytocin hormone. We found that antibiotic treatment increased oxytocin levels in the brain of male C57BL/6 mice, while no changes were observed following Aβ treatment in water- and antibiotic-treated mice. The gut microbiota depletion protocol used in this study is similar to that used by Desbonnet et al. in which no significant alternations in oxytocin levels were found in the hypothalamus of male NIH mice^8^. However, there are differences in the strain of mice and the brain areas. A strong connection exists between brain oxytocin and HPA axis function^73–75^. Brain oxytocin inhibits the basal and stress-induced activity of the HPA axis in male and female rats^73,74^. Although we did not find any significant changes in brain oxytocin levels of antibiotics mice treated with Aβ, a significant correlation was found between corticosterone and oxytocin levels. It is therefore possible that such connections may exist between oxytocin and the HPA axis in depressive-like behaviour following gut microbiota depletion induced by antibiotic treatment in male C57BL/6 mice. Our results cannot directly prove this mechanism and surely additional experiments are needed before firm conclusions can be drawn. Finally, a number of limitations need to be considered. First, we used only male C57BL/6 mice; therefore, it is difficult to assume that the effects of gut microbiota depletion from early adolescence on anxiety- and depression-related behaviours would also be similar in adult females with Alzheimer-like condition. The second limitation is that all animal behaviours were recorded by human but not automated software which may not have the same sensitivity than automated analysis by video tracking system.

## Conclusion

In the current study, we found that antibiotics treatment from early adolescence in healthy mice decreased anxiety-like behaviour in adulthood, while it did not affect depression-like behaviour and HPA axis function in mice. Antibiotic treatment also prevented the development of anxiety- and depression-related behaviours, oxidative stress and HPA axis dysregulation in Alzheimer-induced mice. In addition, antibiotic treatment elevated oxytocin levels in the brain of healthy but not Alzheimer-induced mice. Overall, our findings suggest that gut microbiota depletion from early adolescence might profoundly influence anxiety- and depression-related behaviours, and HPA axis function in mice with Alzheimer-like disease.

## Methods

### Ethics

All procedures were approved by the Research and Ethics Committee of Maragheh University of Medical Sciences (IR.MARAGHEHPHC.REC.1397.008) and complied with ARRIVE guidelines. All methods were also performed under the relevant guidelines and regulations.

### Animals

Thirty-four time-mated female C57BL/6 mice (11–12 weeks-old, 18–21 g weight) were maintained in a room under standard conditions (12 h light–dark cycle; the temperature of 23 ± 1 °C; humidity 40–50%). The breeding procedure was performed as previously described^76^. Briefly, two male and four female mice were placed in a partitioned cage with no physical contact for 3 days. Then, one male and one female mouse were put in a cage together. The next morning (7:00 A.M.), the presence of a vaginal plug was considered a successful mating and gestational day (GD0). Animals were allowed to drink and eat autoclaved water and food ad libitum. Pregnant animals were allowed to deliver naturally their pups (all litters were culled to 6 pups, 3 males and 3 females, per mother) and the day of birth was considered as postnatal day (PD)0. All litters were weaned on PD21 (10–11 g weight).

### Experimental design

**Figure 9:**
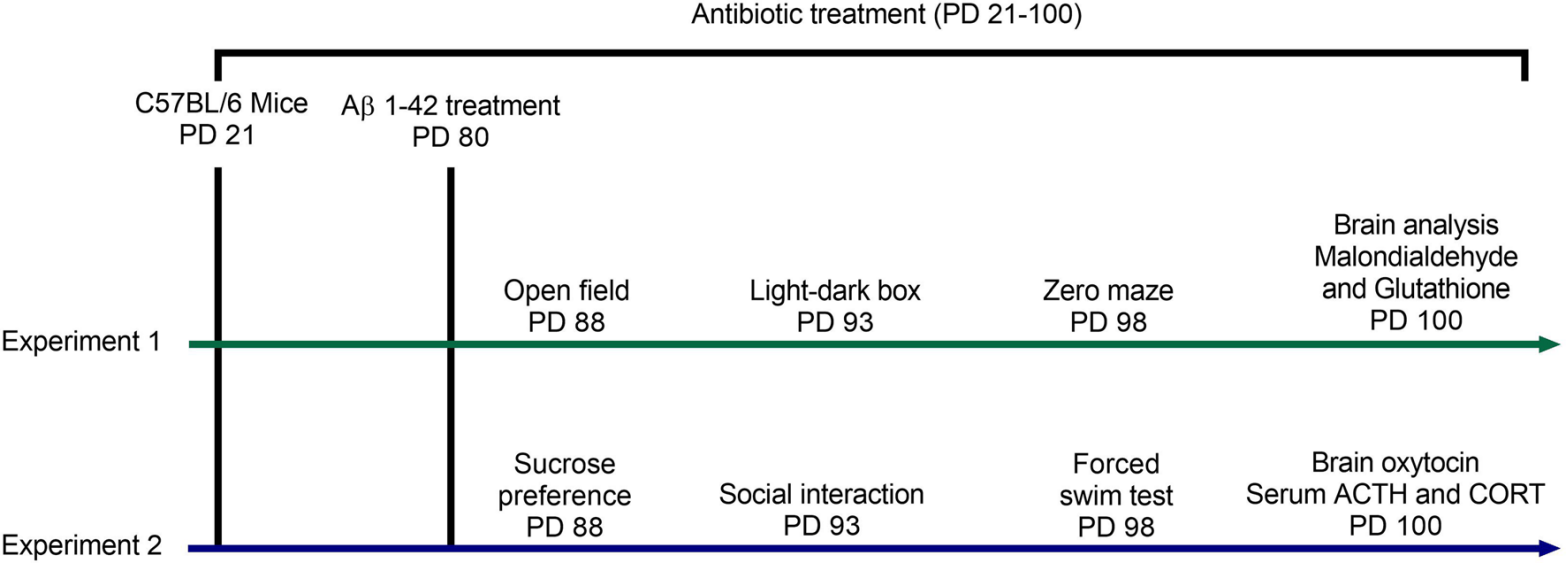
Schematic timeline of the experimental design.

As illustrated in the scheme of the experimental design in Fig. 9, two experiments were performed to evaluate the impacts of gut microbiota depletion from early adolescence on anxiety- and depression-related behaviours in mice with or without Alzheimer-like disease in adulthood (PD88-100). To explore the underlying mechanisms, in experiment 1, we measured malondialdehyde and glutathione in the brain of animals following anxiety tests; in experiment 2, we assessed oxytocin in the brain and ACTH and corticosterone in the serum of animals following depression tests. In each experiment, adolescent male C57BL/6 mice were randomly divided into two main groups (water and antibiotic). One male from each litter was randomly assigned to each main group. On PD 80 (22–25 g weight), each main group was randomly divided into two sub-groups and treated with phosphate-buffered saline or Aβ1–42. A total of 80 male mice were used in this study, and there were 10 mice in each sub-group. Female mice were used in another study. Animals from each sub-group were housed in groups of 5 per cage (42 × 27 × 15 cm).

### Gut microbiota depletion

A combination of different antibiotics (neomycin: 10 mg/ml; ampicillin: 1 mg/ml; metronidazole: 10 mg/ml; vancomycin: 5 mg/ml) and an antifungal drug (Amphotericin-B: 0.1 mg/ml) were used to deplete gut microbiota from early adolescence (PD21) to adulthood (PD100) in mice. This procedure was previously shown to significantly alter neurobehavioral responses in mice and decrease the faecal bacterial DNA load by 400-fold without affecting the animals’ health^5,8^. To avoid the unknown effects of chronic stress following oral gavage, animals received antibiotics or autoclaved water via regular drinking bottles^5,8^. The drug concentration was adjusted according to the average liquid consumption and body weight per cage as previously described by our group^5^. The cages were changed twice a week to prevent the re-establishment of gut microbiota.

### Alzheimer-like disease

To induce Alzheimer-like disease, adult mice (PD80) were anaesthetized by an intraperitoneal injection of pentobarbital (70 mg/kg; Tocris) and placed in a stereotaxic apparatus (Stoelting). Aβ1–42 was dissolved in sterile phosphate-buffered saline (0.1 M; pH 7.4) and aggregated by incubating for 7 days at 37 °C before use^24^. Aβ1–42 (400 pmol/1 μl/mouse) or vehicle was gradually (1 μl/min) administered by intracerebroventricular injections into the right and left lateral ventricle (AP: − 0.1 mm, ML: ± 1 mm and DV: − 3 mm; 0.5 μl/side)^77^. The advantage of this route of administration is the quick distribution of the peptide throughout the brain.

### Behavioural testing

All behavioural tests were videotaped during the light period (between 12:00 and 16:00 h) under the illumination of 75 lux. Then, behavioural parameters were recorded by an expert observer blind to the treatment condition. Animals were kept in the room for at least 1 h before the assessment and tested in an order counterbalanced. At the end of each test session, behavioural apparatuses were carefully cleaned with 70% ethanol. After finishing each behavioural test, the cages were transported back immediately to the colony room.

### Open field

The open-field test was used to evaluate anxiety-like behaviour and locomotor activity in animals (PD88) as previously described^78^. “The apparatus consisted of a box (40 × 40 × 20 cm) with 16 squares (10 × 10 cm; 12-outers and 4-inners). A mouse was placed in the middle of the apparatus and allowed to move for 5 min. The inner zone time and entries were recorded as indices of anxiety-like behaviour. Total line crossings and rears were evaluated as horizontal and vertical activities^79^. A line crossing was defined as all four paws crossing over the line and the rear was recorded when the mouse stood on its hind legs”.

### Light–dark box

Mice (PD93) were exposed to the light–dark box to examine anxiety-like behaviour as previously described^80^. “The apparatus consisted of a rectangular box (length 46 cm, width 27 cm, height 30 cm), which divided into two compartments (light-large: 27 × 27 cm, dark-small: 18 × 27 cm). An open door (7.5 × 7.5 cm) was located in the centre of the partition. The large compartment was open at the top, and the small compartment had a removable black lid at the top. Each mouse was placed at the centre of the light compartment and allowed to explore both compartments for 5 min. The latency to enter the light compartment after the first entry into the dark division, the amount of time spent and the numbers of entries into the light compartment and faecal pellets were recorded”.

### Zero maze

Animals (PD98) were subjected to zero maze to evaluate anxiety-like behaviour as previously described^81^. “A ring-shaped apparatus (diameter 46 cm, width 5.5 cm) was divided into four equal quadrants (two open and two closed) elevated 40 cm from the floor. Each animal was placed into one of the closed quadrants and allowed to explore the apparatus for 5 min. The time spent and the number of entries into the open quadrants, the number of head dips, and the number of stretches attend postures were recorded as indices of anxiety-like behavior”.

### Sucrose preference

The sucrose preference test was used to measure anhedonia-like behaviour in animals (PD88) as previously described^82^. “The sucrose preference test was performed over a 48-h period. No previous food or water deprivation was applied before the test. Mice underwent 3 days of acclimation to the two-bottle choice paradigm (two identical water bottles, both containing tap water, were placed on the cages during acclimation). After 3 days of the acclimation period, each animal was given two bottles, one containing a 2% sucrose solution (A) and the other containing tap water (B). The position of bottles A and B was changed every 12 h to avoid a “side” bias. The amount of the sucrose solution or water consumed was measured by weighing the bottles immediately before and after the test. The sucrose preference was calculated as the percentage of sucrose solution ingested relative to the total amount of liquid consumed. After the test, animals were given free access to water”.

### Social interaction test

The social interaction test was used to assess depression-related behaviour in mice (PD93) as previously described^83^. “Each mouse was exposed to an unfamiliar same-treated mouse. Animals were placed in opposite corners of a square (40 × 40 × 20 cm) arena and allowed to explore for 7 min. The amount of time that the animals spent interacting with each other was considered as social behaviour. The interaction between animals was defined as sniffing (the focal animal establishes contact or near-contact with its nose to the other animals’ body part), following (the focal animal moves/follow the other to maintain a close distance while the other animal is moving around/away), grooming (the focal grooming animal has one or both front paws on top of the other and pulls/licks at its fur), and climbing over each other (the focal animal climbs over the dorsal surface of the other animal)^83^”.

### Forced swim test

Animals were subjected to the forced swim test to assess behavioural despair in mice (PD98) as previously described^84^. “The following procedure was adopted, mice were individually placed into the transparent glass cylinders (height: 25 cm, diameter: 10 cm), filled with water (depth: 15 cm) and maintained at 25 ± 1 °C. The total duration of immobility was recorded during the last 4 min of the 6 min testing period. Each mouse was judged to be immobile when it ceased struggling and remained floating motionless in the water and making only those movements necessary to keep its head above water”.

### Brain and serum analysis

Two days after the anxiety and depression tests, animals on PD100 were anaesthetized by pentobarbital (70 mg/kg; Tocris). A cardiac puncture procedure was used to collect blood into sterile tubes. Blood samples were allowed to clot on an ice-pack for 15–20 min, and then to collect serum the blood samples were centrifuged at 3000 rpm for 10 min^85^. Finally, serums were transferred into sterile tubes and stored at − 20 °C until further processing. To collect brain tissues, after collecting blood, mice were perfused with sterile ice-cold saline (NaCl, 0.9%) to prevent the retention of blood in the brain. Whole brains were rapidly removed, placed into sterile vials and stored at − 80 °C. To collect supernatants, the brain samples were added to 1000 μl homogenizing buffer (TBS plus 0.2% Triton X-100, 2 mM EDTA, PBS 1 mM PMSF, and protease inhibitors) and then this mixture was centrifuged at 15,000*g* for 15 min at 4 °C. The bicinchoninic acid assay kit (Sigma, BCA1) was used to determine the total protein in brain tissue samples. To measure oxytocin (pg/mg-protein; Mybiosource, MBS745701), malondialdehyde (nmol/mg-protein; Sigma, MAK085) and glutathione (µmol/g-tissue; Sigma, CS0260) in the brain tissue supernatants, and ACTH (Abcam Co, ab263880) and corticosterone (Mybiosource, MBS264846) levels in serum were detected using ELISA or specific detection kits according to manufacturers’ instructions.

### Statistics

The statistical package of SPSS (IBM, Version-26) was used to analyze the data (no data were excluded). All data were collected in a blinded manner, and analyzed by a two-way analysis of variance (ANOVA; the results section) followed by Tukey’s HSD test (the legend of figures). Pearson correlation was used to detect the correlations between behavioural and/or biochemical parameters. The data are expressed as mean ± SEM. *P* < 0.05 was considered to be statistically significant.

## Data Availability

Data will be available upon request.
